# A checklist-based method for improving COPD care for the elderly in general practice: study protocol for a cluster randomized controlled trial using electronic health records

**DOI:** 10.1186/s13063-021-05103-0

**Published:** 2021-02-25

**Authors:** Anna Kowalczyk, Izabela Zakowska, Ewa Andrzejewska, Katarzyna Kosiek, Maciek Godycki-Cwirko

**Affiliations:** Centre for Family and Community Medicine, Faculty of Medical Sciences, Medical University of Lodz, Kopcinskiego 20, 90-153 Lodz, Poland

**Keywords:** COPD, Elderly, Exacerbations, Prevalence, Checklist, Randomized controlled trial, Electronic health records

## Abstract

**Background:**

The third most frequent chronic condition, and the fourth most common cause of death, in Poland is chronic obstructive pulmonary disease (COPD). The diagnosis and treatment of COPD is the responsibility of the general practitioner (GP); the GP also serves as gatekeeper, referring patients to the other levels of public health care system when necessary. Undertreatment of COPD can result in a greater frequency of exacerbations and hospitalizations. Elderly patients require special attention due to the increased prevalence of COPD and systemic comorbidities. However, both the occurrence of exacerbations and the quality of life of the patients may be improved by developing and implementing guidelines for practice and ensuring their adherence. This proposal concerns the development of a checklist-based educational program to assist general practitioners in managing COPD patients.

**Methods:**

No less than eighty-four general clinics in the Lodz region, Poland (28 clusters in each of three study arms), will be identified, randomized, and included in the trial. The trial will be based on anonymized data in electronic health records within the national public health care system.

The educational intervention program will consist of GPs in two intervention arms being provided with a COPD management checklist: those in the first intervention arm with receive the checklist once at the beginning, while those in the second with receive it twice. The third (control) arm receives standard care without the checklist.

The study used the International Code of Diseases (ICD)-10 for COPD. The primary aim is to determine the effect of interventions delivered to general practitioners (GPs) in primary health care. These interventions are aimed at decreasing the hospitalization of elderly patients with medical code J-44 (COPD) as the main reason for hospital admission.

**Discussion:**

The results of this trial will be directly applicable to primary care in Poland and add new data to the growing body of evidence regarding interventions aimed at improving chronic illness care.

**Trial registration:**

This trial has been registered with the Clinical Trials Protocol Registration System. Please see in ClinicalTrial.gov identifier (NCT Number): NCT04301505. Registered on 10 March 2020.

**Supplementary Information:**

The online version contains supplementary material available at 10.1186/s13063-021-05103-0.

## Contribution to the literature

- This document describes the design and implementation of a pragmatic study to assess the effectiveness and implementation of a strategy based on a structured checklist in primary care.
- The impact of the checklist on care in COPD will be determined using Big Data methods.
- The clinical effectiveness of repetition of COPD checklist exposure in primary care will be assessed.

## Introduction

### Background

Caring for patients with chronic obstructive pulmonary disease (COPD) presents a substantial challenge for general practitioners (GPs). COPD is a non-reversible lung condition characterized by shortness of breath, chronic cough with sputum production, emphysema, and systemic pulmonary inflammation.

Although data on its prevalence in the general population is limited, the true prevalence of COPD among the Danish population is believed to be 9% (95% CI 8–10%), with the highest prevalence observed among current smokers (23%) and former smokers (17%) as well as among seniors (total = 18%; men = 21%; women = 15%) [1].

In Australia, approximately 5% of patients in general practice were found to suffer from COPD; this number rises to approximately 10% among patients aged 65 years or above [2].

Although the scope of the problem is recognized in Poland, relatively little concrete research data exists, especially concerning elderly patients in primary care. It is estimated that the prevalence of COPD is approximately two million out of a population of about 38 million; this places COPD as the third most widespread chronic condition in the country and the fourth most common cause of death [3].

However, international studies indicate that the prevalence of COPD in Poland may be considerably higher than previously anticipated (10% of the examined population) [4].

In Poland, almost everyone registered with the national public health care system (NFZ) is also registered with a GP. The GP acts as gatekeeper, referring patients with diagnostic or treatment problems to the other levels of the public health care system. However, it is possible that some mistakes may occur with diagnosis and treatment. Under-treatment can result in unnecessary symptom burden, impaired COPD control, and more frequent exacerbations and hospitalization, while over-treatment may lead to increased healthcare costs and potential iatrogenic effects [5].

Elderly patients are at greatest risk of developing COPD and its components: emphysema, chronic bronchitis, and bronchiectasis. Bacterial and viral infections also play a role in acute exacerbations of COPD, and elderly patients are at greater risk of infection by antibiotic-resistant bacteria [6].

To introduce effective interventions and inform efforts for health resource allocation, it is necessary to understand the features of COPD in older patients. Non-smoking seniors tend to demonstrate a greater prevalence of COPD and a higher rate of systemic comorbidities. In addition, acute exacerbations in older patients tend to have a poorer outcome, reflected in an increased rate of hospitalization, longer stays, and increased re-hospitalization and mortality rates. Seniors are also more likely to experience impaired cognitive functions and problems affecting the hand joints, thus impairing the effectiveness of inhaled medications and the outcome of care. Even for those who are competent at using inhalers, the evidence for their efficacy in older patients is uncertain [7].

A Danish study indicates substantial room for improvement in GP clinics [8].

Other studies indicate that even the routine implementation of community health assessment and improved planning measures can lead to improved health outcomes [9]. The annual healthcare costs for primary care for these patients are estimated to be approximately 53.6 million euros. Various interventional approaches have been used [10].

COPD exacerbations significantly impact health-related quality of life and disease progression, and healthcare costs associated with severe exacerbation-related hospitalization range from $7000 to $39,200 in the USA. However, the timely and appropriate application of maintenance pharmacotherapy, particularly dual bronchodilators for maximizing bronchodilation, can significantly reduce exacerbations, and multidisciplinary disease-management programs including pulmonary rehabilitation, follow-up appointments, aftercare, inhaler training, and patient education can reduce hospitalizations and readmissions for patients with COPD [11].

However, few physicians are aware of practice guidelines and continuing medical education (CME) programs [12]. Current guidelines are still poorly adhered to by GPs, and generally, little is known of the role of specific non-clinical interventions in facilitating GP care for COPD patients. To remedy this, the EU-funded TICD project, a cluster randomized controlled trial (RCT), was performed to identify possible determinants of COPD care and tailored interventions to facilitate better implementation in general practice [13]. Such intervention appears feasible [14].

The present study will identify the most effective method for delivering an educational intervention to general practitioners based on a simple consensus process.

This protocol has been written in line with the SPIRIT standard [15].

### Objectives

1. To determine the effect of intervention aimed at decreasing the hospitalization of elderly patients with J-44 as the main reason for hospital admission, compared to those receiving usual care
2. To optimize the management of elderly COPD patients
3. To examine whether intervention 1 and intervention 2 are effective, pragmatic, and feasible within the primary care setting

### Trial design

The trial is planned as a 1-year, three-arm, pragmatic, cluster randomized trial (CRCT). It will compare the effectiveness of interventions in two arms (single intervention and double intervention) at reducing the hospitalization of seniors in primary care with the J-44 code as a main reason for admission, compared to those experiencing usual care.

## Methods/design

### Study setting

Eighty-four GP clinics in the Lodz voivodship in Poland will be identified at baseline using data from the National Health Fund’s (*Narodowy Fundusz Zdrowia*, NFZ) electronic medical records (EHR). They will be randomly selected using the following criteria: clinics with at least 30 patients per clinic, aged 65 years and older with COPD. Patients with COPD will be identified by the ICD-10 code J-44 in NFZ electronic medical records; exacerbations will be defined as cases hospitalized with the J-44 code as the main reason for admission.

### Observation period

**Fig. 1:**
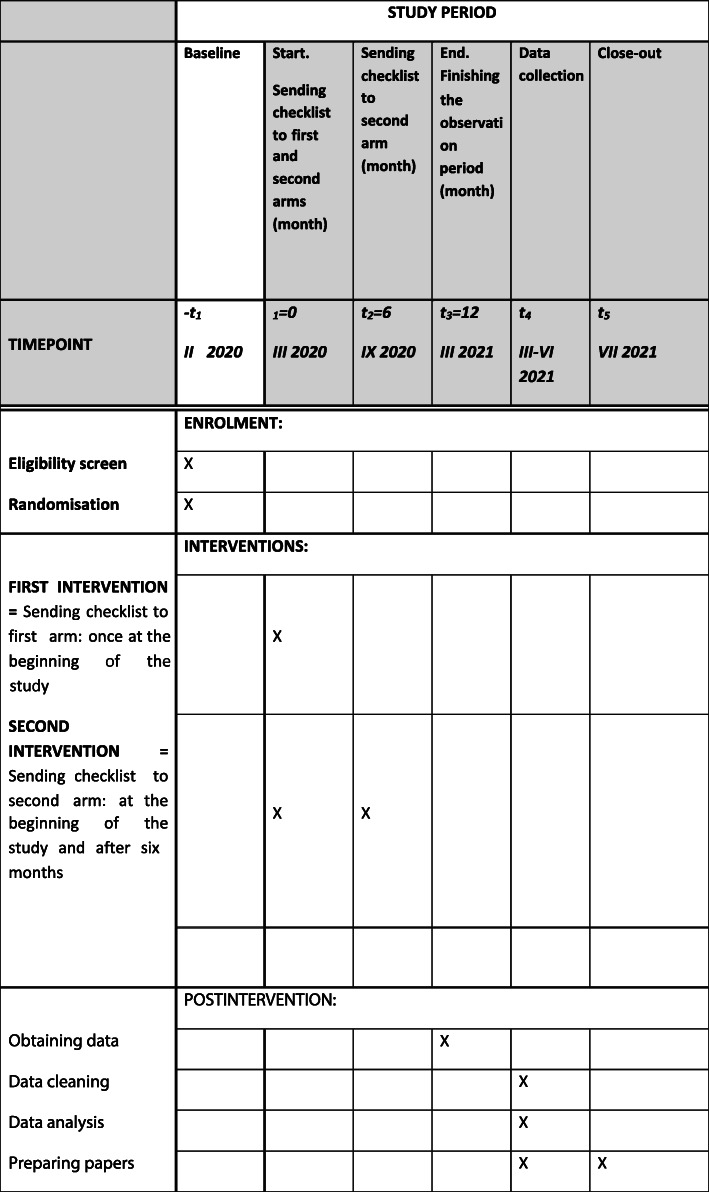
The schedule of the study

The study will include data for 1 year, from March 2020 until February 2021. Data extraction and analysis will take place from March until June 2021 (see Fig. 1).

The endline data for the clinic and patients will be collected after 1 year. We will consider additional data collections from other time points based on the development of the COVID situation.

### Sample size

A minimum number of 84 GP clinics with at least 30 J-44 patients per clinic is required to provide a sufficient sample size for a three-arm cluster randomized controlled trial: 84 clusters and at least 2520 subjects are needed (28 clusters in each study arm) to detect a difference of 0.026 between mean event rates of hospitalization with ICD-10 code J-44 as the main reason for admission. This assumes a 25% reduction in total number of events after the intervention in the first arm (receiving checklist once) and 50% reduction in total number of events after the interventions in the second arm (receiving checklist twice) compared to the control group not receiving any intervention (standard care). It is also assumed the tests will have 80% power, a two-tailed alpha of 0.05, and intra-cluster correlation of 0.01.

Data collected from electronic health records in 2016 indicate that the mean event rate of “hospitalized with ICD-10 code J-44 as the main reason for admission” in the control group is 0.105, and the standard deviation is 0.306. The unadjusted sample size was calculated for differences between three means for one-way ANOVA.

To compare the individuals hospitalized with the J-44 code in the three arms after 1 year, the difference between the mean event rates of the three groups was calculated according to Donner and Klar [16].

A standard sample size formula was used to calculate the initial unadjusted sample size requirements, followed by appropriate adjustment for clustering by general practice according to Campbell et al. [17], with expected small clustering effect (ICC = 0.01) [18, 19].

To prevent loss to follow-up among practices/participants, an enlarged overall sample size will be deemed appropriate to ensure adequate power.

### Randomization

The clinics will be randomized without repeating by a Data Scientist (not involved in the trial) using a computer system. Each GP clinic will be allocated randomly to one of three equal-sized arms: two intervention arms and a control arm by Data Scientist.

The clinics from the intervention arms will find out that they are enrolled when they receive the letters with interventions. The letters will be sent simultaneously. The control arm will only use usual care and has no intervention.

### Blinding

Participants in this trial will be anonymized rather than blinded. Because of the nature of the interventions, it is not possible to blind the GP participants in the clinics. Outcome assessment will not be blinded as the researchers will be aware of practice arm allocation. The Data Scientist will not be blinded, because she will be independent of the research team.

### Interventions

The educational intervention program will consist of providing GPs in two intervention arms with a COPD management checklist (Additional file 1).

In the first intervention arm, the checklist will be delivered at the beginning of the study (March 2020); in the second arm, it will be delivered at the beginning of the study and repeated after 6 months. Clinics that are randomized to the control arm will not receive the COPD management checklist, and GPs will treat all patients according to standard care.

The checklist was developed by authors on the basis of GOLD guidelines [20] and the 2015 Physician Quality Reporting System (PQRS) Implementation Guide Centers for Medicare & Medicaid Services [21].

### Outcomes

*The primary outcome of the study* will be the effect of the interventions on the proportion of “hospitalization with the J-44 code as a main reason for admission,” and the proportion of deaths of elderly COPD patients, registered within practices after 1 year.

*Primary outcome at the patient level* will be assessed as the proportion of cases between the three arms (intervention 1, intervention 2 and control) at the end of the 1-year study period. Proportion of cases (event rates) of binary variables of exacerbation outcome will be assessed based on “hospitalization with the J-44 code as a main reason for admission,” and the proportion of deaths of elderly COPD patients.

The effect of the educational intervention will be evaluated using logistic 2-level regression analysis, based on odds ratios (OR) and 95% confidence limits.

We will examine the association between intervention and COPD exacerbation, using a case-control approach. Cases will be selected among COPD exacerbation after 1 year. Control subjects will be selected among COPD patients.

*Secondary outcome at the patient level* will be the proportion of the control and intervention arms regarding the specific short- and long-acting drugs prescribed after 1 year.

### Data collection methods

Patient data will be obtained from Big Data databases, such as the patient electronic health record system from the National Health Fund (NFZ).

### Data management

Depersonalized data will be obtained and subjected to quality control and cleaning.

The structure of the obtained data will be hierarchical. Individual patient data will be anonymized. Patients will be nested within PC clinics. The approach to missing data will be determined once the data is obtained.

Big Data cleansing process will be performed according to SAS Data Management Methodology. This methodology covers a step-by-step process, and it covers tasks such as data management, data quality, data integration, data migrations, and master data management.

Study variables: demographic and characteristics will include age; gender; residence code; “hospitalized, with the J-44 code as the main reason for admission”; death; specific short- and long-acting medication prescribed; and number of GPs.

### Statistical methods for analysis

Post-intervention analysis will be performed.

The following data will be analyzed in the post-intervention patients: hospitalization with the J-44 code as the main reason for admission, and use of medication. The analysis will be based on multilevel regression models.

#### Categorical predictors

Educational interventions after 1 year in two arms, and an arm with standard care (control), age and gender.

#### Test statistics for primary outcome and dependent variable

The effect of the educational interventions will be evaluated using two-level logistic regression models with a dependent binary variable (case-control, cross-sectional analysis), with patients nested within practices.

Data regarding hospitalization will be collected directly from patient electronic health records held by the National Health Fund (NHF—the state health insurance system). Patients registering exacerbation, defined as “hospitalization of patients with the J-44 as a main reason for admission,” will be classed as cases. Control subjects will be collected from electronic health records held by the NHF, defined as COPD (J-44), age over 65, with low treatment cost, and no constantly reimbursed drugs. Data will be collected after the 1-year study period (cross-sectional) within the Lodz voivodeship.

Descriptive statistics and logistic regression will be employed. The level of statistical significance will be *p* < 0.05.

## Discussion

Our methods will be used to quantitatively evaluate the impact of the proposed educational program on reducing COPD exacerbations among COPD patients presenting at primary care clinics compared with existing standard care.

Its main limitations relate to the specificity and sensitivity of the COPD coding, gaps in databases, and short period of observation. In addition, some selection bias may exist in the study, since small practices will be excluded. Furthermore, the COVID epidemic could also distort the effects of the intervention and influence the results. Finally, the participation of the National Health Fund and the sending of letters may be disrupted.

Nevertheless, we believe that the results will be directly relevant and applicable to primary care in Poland. If the implementation is effective, then wide-scale application would be warranted. We will consider additional data collection time points to extend further analysis to account for the impact of COVID.

## Trial status

The trial is currently in the interventions phase.

ClinicalTrial.gov identifier: NCT04301505. Registered on 10 March 2020, https://clinicaltrials.gov/ct2/show/NCT04301505

Protocol Version 9: 16 January 2021.

Date recruitment began: March 2020.

Estimate of the date when recruitment will be completed: March 2021.

## Supplementary Information

**Additional file 1.** GP COPD checklist.

## Data Availability

Not applicable.

## Abbreviations

CME: Continuing medical education
COPD: Chronic obstructive pulmonary disease
CRCT: Cluster randomized controlled trial
ICD-10: International Classification of Diseases
NFZ: National Health Fund
GP: General practitioner
PC: Primary care
EHRs: Electronic health records
TICD: Tailored Implementation for Chronic Disease
