# Supplementary material for: A checklist-based method for improving COPD care for the elderly in general practice: study protocol for a cluster randomized controlled trial using electronic health records
Source: Trials. 2021 Feb 25;22:161. doi: 10.1186/s13063-021-05103-0 (PMC7905541; doi:10.1186/s13063-021-05103-0)
Supplement: Supplementary file 1 — Additional file 1. GP COPD checklist. [file 13063_2021_5103_MOESM1_ESM.docx]

GP COPD checklist

I. Check diagnosis and patient's condition

Classification of COPD

**
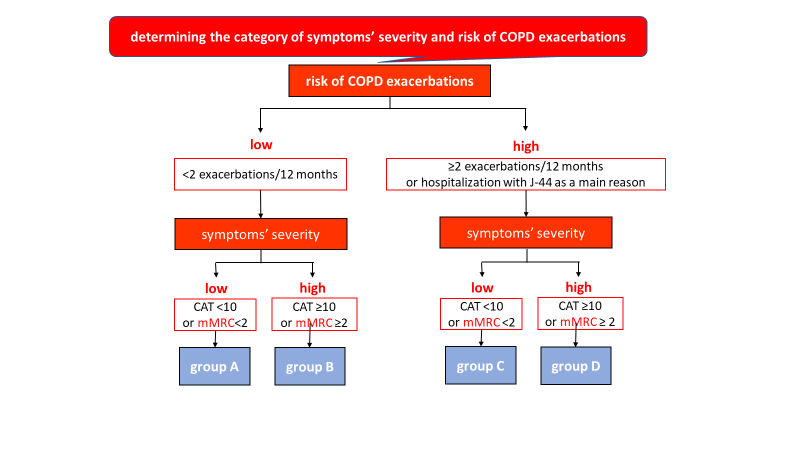
**

II. Patient check

1. Smoking cessation → stop smoking
2. Vaccinations (i.e. influenza, pneumonia) → advise vaccination
3. Importance of physical exercises → advise physical exercises
4. Medication adherence (i.e. knows how short and long acting medications work)

→ advise proper use

1. Proper use of inhalers → check the technique, instruct
2. Recognition and treatment of exacerbations → advise when and where ask for help
3. Follow-up visits → schedule next visit

III Quality Measures Check

1. Spirometry evaluation: percentage of patients aged ≥18 years with a diagnosis of COPD who had spirometry results documented.
2. Inhaled bronchodilator therapy: percentage of patients aged ≥18 years with a diagnosis of COPD and who have an FEV1/FVC < 60%, have symptoms and who have been prescribed an inhaled bronchodilator.
3. Influenza immunization: percentage of patients seen during influenza period who received an influenza immunization.
4. Pneumonia vaccination: percentage of patients aged ≥65 years who have ever received a pneumococcal vaccine.
5. Documentation of current medications in the medical record: percentage of visits of patients ≥18 for whom a list of current medications was reported.
6. Tobacco use: Percentage of patients ≥18 years of age who were screened for tobacco use within 24 months and received counseling intervention on smoking cessation if identified as tobacco user

**References:**

1. Global Initiative for Chronic Obstructive Lung Disease (GOLD). Global Strategy for the Diagnosis, Management, and Prevention of Chronic Obstructive Pulmonary Disease. Available at: https://goldcopd.org/wp-content/uploads/2018/11/GOLD-2019-POCKET-GUIDE-DRAFT-v1.7-14Nov2018-WMS.pdf

2. Postępowanie w POChP – co nowego w wytycznych Global Initiative for Chronic Obstructive Lung Disease (GOLD) 2019 dr n. med. Filip Mejza https://www.mp.pl/pulmonologia/artykuly-wytyczne/pochp/202174,postepowanie-w-przewleklej-obturacyjnej-chorobie-pluc

3. Centers for Medicare & Medicaid Services. 2015 Physician Quality Reporting System (PQRS) Implementation Guide. Available at: www.cms.gov. Accessed 4/21/15.
